# Segmental duplications: evolution and impact among the current Lepidoptera genomes

**DOI:** 10.1186/s12862-017-1007-y

**Published:** 2017-07-06

**Authors:** Qian Zhao, Dongna Ma, Liette Vasseur, Minsheng You

**Affiliations:** 1 State Key Laboratory for Ecological Pest Control of Fujian/Taiwan Crops and College of Life Science, Fujian Agriculture and Forestry University, Fuzhou, 350002 China; 2 Institute of Applied Ecology, Fujian Agriculture and Forestry University, Fuzhou, 350002 China; 3 Fujian-Taiwan Joint Centre for Ecological Control of Crop Pests, Fujian Agriculture and Forestry University, Fuzhou, 350002 China; 4 Key Laboratory of Integrated Pest Management for Fujian-Taiwan Crops, Ministry of Agriculture, Fuzhou, 350002 China; 5 Department of Biological Sciences, Brock University, 1812 Sir Isaac Brock Way, St. Catharines, ON L2S 3A1 Canada

**Keywords:** Segmental duplications, Lepidoptera, Evolution

## Abstract

**Background:**

Structural variation among genomes is now viewed to be as important as single nucleoid polymorphisms in influencing the phenotype and evolution of a species. Segmental duplication (SD) is defined as segments of DNA with homologous sequence.

**Results:**

Here, we performed a systematic analysis of segmental duplications (SDs) among five lepidopteran reference genomes (*Plutella xylostella*, *Danaus plexippus*, *Bombyx mori*, *Manduca sexta* and *Heliconius melpomene*) to understand their potential impact on the evolution of these species. We find that the SDs content differed substantially among species, ranging from 1.2% of the genome in *B. mori* to 15.2% in *H. melpomene*. Most SDs formed very high identity (similarity higher than 90%) blocks but had very few large blocks. Comparative analysis showed that most of the SDs arose after the divergence of each linage and we found that *P. xylostella* and *H. melpomene* showed more duplications than other species, suggesting they might be able to tolerate extensive levels of variation in their genomes. Conserved ancestral and species specific SD events were assessed, revealing multiple examples of the gain, loss or maintenance of SDs over time. SDs content analysis showed that most of the genes embedded in SDs regions belonged to species-specific SDs (“Unique” SDs). Functional analysis of these genes suggested their potential roles in the lineage-specific evolution. SDs and flanking regions often contained transposable elements (TEs) and this association suggested some involvement in SDs formation. Further studies on comparison of gene expression level between SDs and non-SDs showed that the expression level of genes embedded in SDs was significantly lower, suggesting that structure changes in the genomes are involved in gene expression differences in species.

**Conclusions:**

The results showed that most of the SDs were “unique SDs”, which originated after species formation. Functional analysis suggested that SDs might play different roles in different species. Our results provide a valuable resource beyond the genetic mutation to explore the genome structure for future Lepidoptera research.

**Electronic supplementary material:**

The online version of this article (doi:10.1186/s12862-017-1007-y) contains supplementary material, which is available to authorized users.

## Background

Segmental duplications (SDs) are DNA fragments with near-identical sequences that are greater than 1Kb [1]. They have been recognized as important mediators of gene and genome evolution, and are considered the origins for gene gain, functional diversification, and gene family expansion [1, 2]. The outcomes of a gene duplication event may lie on lineage-specific selection. In this situation, the new gene copy has the opportunity to acquire novel or modified functions or become non-functional [3, 4]. These new copies are often important for the adaption of the species to certain environments [2]. SDs can lead to various types of genome rearrangements [5] and other genome structural changes between and within species [6–8].

Characterization and annotation of SDs are important for understanding the structure and evolution of a genome and have been explored in many organisms’ whole genomes [9–15]. Few systematically comparative analyses of SDs however have been performed until now. The most important example is primate genomes, used to understand the pattern and rates of SDs during hominid evolution [6]. Here, we performed the comparative analysis of SDs in the whole genomes of five Lepidoptera insects, diamondback moth (*Plutella xylostella*)*,* Monarch butterfly (*Danaus plexippus*), silkworm (*Bombyx mori*), Carolina sphinx moth (*Manduca sexta*), and postman butterfly (*Heliconius melpomene*), to understand the roles of SDs during the evolution of Lepidoptera. Our analysis revealed that duplication activities varied in terms of number of base pairs or events among these different species. The marked difference of transposable elements (TEs) content in the flanking regions of SDs among these species of Lepidoptera suggested various formation mechanisms of SDs. Our functional analysis of the SDs indicated that gene families embedded in the SDs were different among the five genomes and these gene families may be related to species-specific adaptive evolution.

## Methods

### Data sources

The five Lepidoptera insect species, *P. xylostella*, *B. mori*, *D. plexippus*, *M. sexta*, and *H. melpomene*, were used to construct the SD map. The genome and predicted transcripts of diamondback moth was downloaded from DBM database (http://www.insect-genome.com/) [16]. The other genomes resources of Lepidoptera insects were downloaded from SilkDB (http://silkworm.genomics.org.cn/) [17], Heliconius Genome Project (http://www.butterflygenome.org/) [18], MonarchBase (http://www.insect-genome.com/) [19] and Carolina sphinx dataset (ftp://ftp.bioinformatics.ksu.edu/pub/Manduca/OGS2/).

## Computational analysis of lepidoptera segmental duplications

We used the Whole-Genome Assembly Comparison (WGAC) method to detect the segmental duplications in the five Lepidoptera species. The insect genomes were first masked at 15% divergence level from transposable elements (TEs), high-copy repeats or simple sequence repeats (SSR) using RepeatMasker (Smit and Green http://www.repeatmasker.org/, version 4.0.6). We then used silkworm TE dataset [20] as repeat database to re-run the RepeatMasker to mask as much TEs as possible. All these repeats were deleted from the sequences and the remaining genome sequences were used to perform BLASTN searches against themselves with reduced affine gap extension parameters, which allowed gaps up to 1000 bp and e value (1e^−20^).

After discarding self-alignments, the repeat sequences were reinserted back into these alignments. These seed alignments were subsequently used as queries to search against the unmasked genome using BLASTN, which generated accurate alignment statistics. Considering the high rate of heterozygosity of these Lepidoptera species (except silkworm, which has a long history of domestication and inbreeding) [16, 18, 21], we conservatively lowered the identity threshold to 75% for alignments in order to capture more divergent SDs than under the 90% usual threshold. Selected alignments were those with a length longer than 1 kb and identity higher than 75%.

## Gene content and functional annotation

Gene content of segmental duplications was accessed using the GFF files obtained from the dataset above (see data sources). We also assessed whether the molecular function, biological process, and pathway terms were over-represented in SDs using Blast2Go [22]. For each SD, we computed an expected number of genes for different biological processes based on their curated representation in the reference genome. The statistical significance of the functional GO Slim enrichment was evaluated using the Fisher’s exact test (*p* < 0.05).This analysis showed the GO terms that were significantly enriched among genes within SDs. Pfam was also used to annotate the function of the genes in the SDs [23].

## RNA-seq analysis

We collected the RNA-seq data from published sources to access the gene expression level within and outside SDs regions. These data included different tissues or different developmental stages of diamondback moth [16], silkworm [24] and Carolina sphinx moth [25]. All the reads were mapped back to its genome using TopHat [26]. The expression abundance (RPKM) was calculated using CuffLinks [27]. The expression levels were assessed as Log_10_^(RPKM)^. Gene expression levels within and outside SDs regions as well as the variables were compared using a T-test with a Bonferroni correction.

## Results and discussion

### Segmental duplication maps among different Lepidoptera species

Using WGAC, we developed segmental duplication maps for each of the five Lepidoptera species’ genomes (Table 1). SD contents greatly varied among the five Lepidoptera species, ranging from 1.2% in *Bombyx mori* to 15.2% in *Heliconius melpomene* (Table 1, Additional file 1: Table S1). SDs with highest identity (≥90%) was the majority (ranging from 80% in *M. sexta* to 93% in *D. plexippus*) (Table 1). Based on our analysis, duplications varied in size from 5.6 Mbp in silkworm to 43 Mbp in *P. xylostella*. *P. xylostella* and *H. melpomene* showed the highest number of duplications (Table 1) suggesting that their genomes could be unstable or capable of tolerating extensive levels of variation. For example, in human, segmental duplications play an “expanding” role in genomic instability [28].

**Table 1:** Characterization of the SDs of the five Lepidoptera species

Species	P. xylostella	M. sexta	H. melpomene	*D. plexippus*	*B. mori*
Total number of SDs	21,369	11,141	23,942	10,799	3667
Number of SDs with 90% identity	18,064	8892	21,572	10,070	3221
Number of SDs with 80-90% identity	3204	2171	2239	668	416
Number of SDs with 75-80% identity	99	78	127	60	5
Total (Mb)	43	19.1	40.5	23.5	5.6
% of genome	11	5.2	15.2	9.9	1.2
Number of genes	2235	1040	1453	1564	332
% of genes	12.4	6.8	11	10.3	2.3

**Fig. 1:**
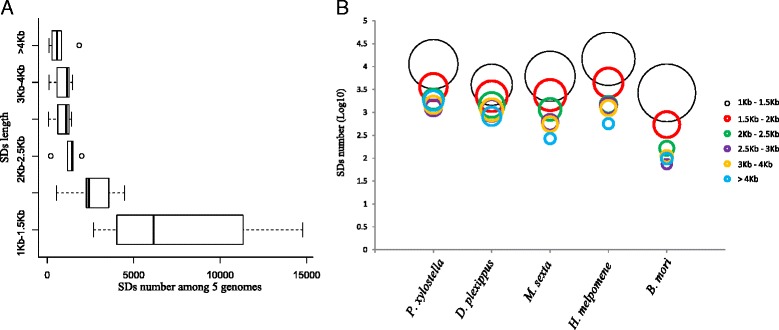
SDs length distributions among 5 Lepidoptera species. **a** Boxplots showing the mean and range of the numbers of SD for each SD length category for all five Lepidoptera species combined. Most SDs were found in the 1–1.5 kb length category. **b** Number of SDs of different length categories for each species. The size of the circle represents the proportion of SDs

The analysis of the length of SDs in all five species indicated that the Lepidoptera genomes were significantly poor in large blocks (>4 Kb) (T-test, *P* < 0.025; Fig. 1). This is consistent with SDs data reported in *Drosophila* genome (Fiston-Lavier et al. 2007) and silkworm genome [29, 30]. The number of SDs in Lepidoptera decreased along with the increase in SDs length (Fig. 1a) and this was true for all five species (Fig. 1b). Eichler [31] has suggested that SDs in invertebrates are much smaller in length than in vertebrates. These differences probably reflect some evolutionary constraints imposed by the smaller size of the invertebrate genome [32].

We use RepeatMasker (Smit and Green http://www.repeatmasker.org/, version 4.0.6) to mask the transposable elements (TEs; masked at 15% divergence level), high-copy repeats or simple sequence repeats (SSR). Then silkworm TE dataset [20] was used as repeat database to rerun the RepeatMasker to mask as much TEs as possible. Thus, we used different repeat databases to mask the target genomes. The result showed that almost 22.6% of the silkworm genome was masked while 2.05% - 4.97% of other Lepidoptera genomes were masked (*P. xylostella*: 3.12%, *D. plexippus*: 2.05%, *M. sexta*: 4.97% and *H. melpomene*: 2.25%). Osanai-Futahashi et al. [33] have shown that TEs are enriched in the genome of silkworm and TEs may play important roles during the domestication of silkworm [34]. Thus, the high proportion of SDs in *H. melpomene* may result from some TEs left in the genome.

### Comparative analysis of duplication maps among five Lepidoptera species

**Fig. 2:**
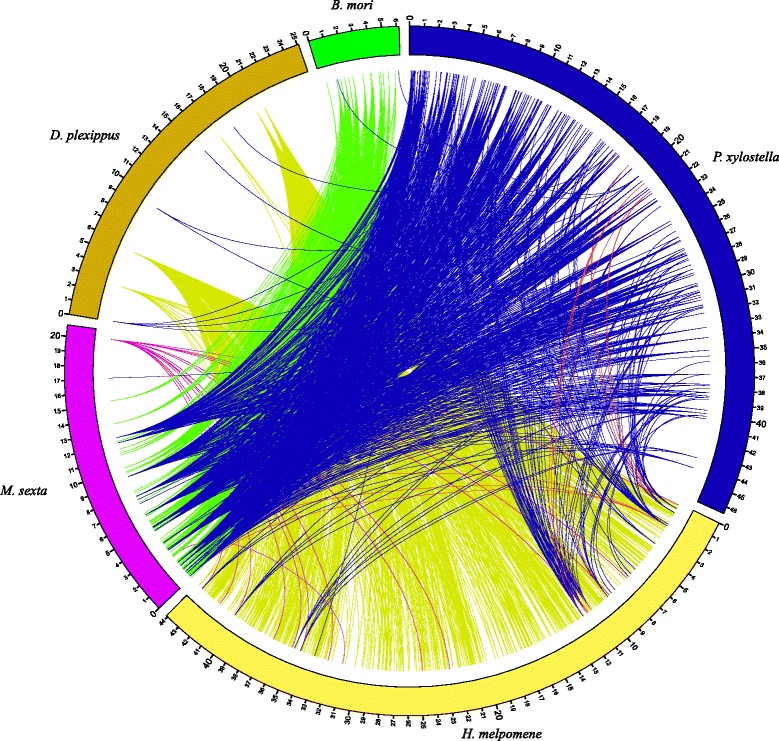
Duplication map comparison of five Lepidoptera species. SDs regions in each Lepidoptera species and their paralogous regions in other four genomes were shown. Different colors represent different insects. Only the best alignments were listed

**Fig. 3:**
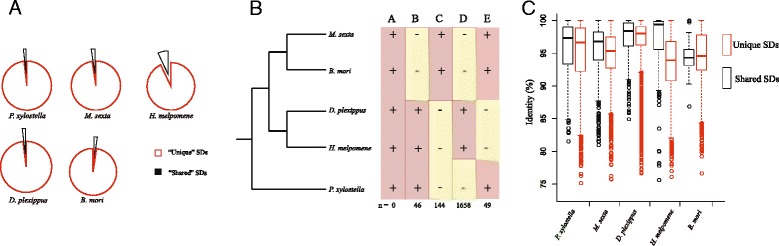
“Shared” and “Unique” SDs among 5 species. **a** “Shared” and “Unique” SDs in each genome. *Red* represents “Unique” SDs while *black* represents “Shared” SDs. **b** SDs classification of the five species based on the existing SDs (marked as “+”) and absence SDs (marked as “-”). We classified the SDs into five groups to analyze evolutionary history of SDs in Lepidoptera genome. Group A are the potential ancestral SDs events. Group B showed the SDs that were only lost in Noctuoidea and Bombycoidea while Groups C, D and E showed regions where duplication patterns were inconsistent with the generally accepted phylogeny. Such a scenario could arise as a result of a de novo origin of SDs or as a result of deleted events, which might have played a role in lineage-specific evolution. “n” represents the number of group A, B, C, D or E. **c** Identity comparison between “Shared” and “Unique” SDs in the genomes of the five Lepidopteron species. The identity of “Shared” SDs is marked as *black* while the identity of “Unique” SDs is marked as *red*

We further characterized each SD as “unique” or “shared”, depending on whether they exist in only one or multiple genomes. The comparative SD maps revealed that most of the segmental duplications were “unique” SDs (Fig. 2). For example, the number of shared SDs among the five Lepidoptera species varied from 83 in *B. mori* (e.g. 83 SDs from *B. mori* shared with other Lepidoptera genomes) to 1817 in *H. melpomene* (e.g. 1817 SDs from *H. melpomene* shared with other Lepidoptera genomes) (Fig. 3a).

Butterflies (*D. plexippus* and *H. melpomene*) shared more SDs with each other than with the other species (Fig. 2) indicating their closer relationship. Silkworm and Carolina sphinx moth also shared more common SDs than with the other species, indicating their close relationship. These results are consistent with the phylogeny of Lepidoptera published by Regier et al. [35].

Based on the phylogeny of Lepidoptera [35], it was possible to assess the origins of some SDs within specific lineages and ancestral events of SDs. Since segments might have mutated after divergence, we attempted to map duplication events onto the phylogenetic tree using reconciliation method (software like NOTUNG). However, based on the blast search analysis, we found that the “unique” SDs could not find the homologous sequences in other Lepidoptera species. We had two speculations to explain this result: (1) The segments might have mutated after duplications or (2) SDs arose after the divergence of each lineage. In the first situation, all the copies should have mutated and evolved rapidly, resulting in sequence variation being too high to find a blast hit in other genomes. If so, it would be difficult to trace the ancestral sequences onto the phylogenetic tree using reconciliation method such as Notung. Thus, we classified the SDs into five groups (Fig. 3b) and focused on analyzing evolutionary history of SDs in Lepidoptera genome. To identify the potential ancestral SDs events, we initially focused on shared duplications among all five species (Group A) but none were identified (Fig. 3b),suggesting that the original SDs might have been lost early during the evolution of Lepidoptera or the origins of the SDs are along with the speciation of the Lepidoptera. The second situation would lead to some SDs that only exist in one or a few genomes.

We then analyzed the SDs that were only lost in Noctuoidea and Bombycoidea (Group B, Fig. 3b). There were 46 cases found in this group. As part of our comparative analyses, we also found some regions where duplication patterns were inconsistent with the generally accepted phylogeny (Groups C, D, and E; Fig. 3b). Such a scenario could arise as a result of a de novo development of SDs or as a result of deleted events, which might play a role in lineage-specific evolution. Groups C and D (Fig. 3b) were more common than the other groups due to their closer relationship with species based on the evolution history (Fig. 3b). The previous study of Marques-Bonet et al. [6] reports that humans share a greater number of SDs with chimpanzees than macaque or orangutan. Only 49 common SDs were lost in *D. plexippus* and *H. melpomene* (Group E). Since the time of Lepidoptera speciation is relatively long, we cannot test the complete phylogeny of SDs, and a greater number of sequenced Lepidoptera genomes would be necessary to elucidate this aspect.

We speculated that the “shared” SDs between species might represent the “ancestral sequences” as they remained conserved in the genomes during the evolution of Lepidoptera. To test this speculation, we analyzed the alignment identity of “shared SDs” and “unique SDs” and found that “shared SDs” had significantly higher identity comparing with the “unique SDs” (*P* < 0.01, T-test), except for silkworm (Fig. 3c). The results indicated that “shared SDs” might be more conserved than the “unique SDs”. Silkworm has diverged from the other species due to its domestication and inbreeding history leading to extremely low level of heterozygosity [36]. We compared the SDs in silkworm with the artificial selected regions that were identified in Xia et al. [36] and found that eight SD regions overlapped with the artificial selected regions, suggesting that these SDs may be related with the silkworm domestication. However, none of these eight regions were “shared SDs”, which also indicated that these unique SDs may be involved in the lineage-specific domestication. We also tested the difference of variance between “shared” and “unique” SDs (Fig. 3c) and showed that in *P. xylostella, D. plexippus* and *H. melpomene*, the differences were not significant (*p* = 0.05755; *p* = 0.5304 and *p* = 0.6278, respectively). Only *B. mori* and *M. sexta* showed significant differences (*p* = 1.218e-05 and *p* = 0.03909).

### Sequence properties of the SDs in the five studied species

The analysis and comparison of the composition of genes in the SDs among the five Lepidoptera species showed that 2235, 1036, 1453, 1564 and 332 putative genes could be identified in *P. xylostella, M. sexta, H. melpomene, D. plexippus* and *B. mori* respectively (Table 1, Additional file 2: Table S2). Most of the segmental duplication intervals identified contained gene duplicates, ranging from 58% in silkworm to 94% in *H. melpomene* (Additional file 3: Table S3)*.* We further characterized the genes as “shared” or “unique” based on whether they were located in the “shared SDs” or “unique SDs”. The results showed that most of the genes belonged to the “unique” genes, with only 31, 26, 13, 6, and 3 genes belonging to “shared” genes in *P. xylostella, M. sexta, D. plexippus, H. melpomene*, and *B. mori*, respectively. These results suggested that most of the genes in SDs could play different roles in different species. We hypothesized that these genes might be involved in lineage-specific evolution and particular gene classes might be overrepresented in the SDs.

To test the hypothesis, we used Gene Ontology (GO) to annotate all the genes and showed that each species had different GO enrichments and gene families (Table 2; Additional file 4: Table S4). In *P. xylostella*, 25 proteins were identified such as serine-type endopeptidase activity (GO: 0004252), structural constitute of cuticle (GO: 0042302), and nucleic acid binding (GO: 0003676) (Table 2). Based on previous study of differential expression in response to host-plant on Swedish comma, *Polygonia c-album*, these genes may be related to host-feeding [37, 38]. Thus, we suggested that the genes in SDs of diamondback moth might be related with its host-feeding behavior.

**Table 2:** GO enrichment for some proteins within the SDs regions among the five Lepidoptera species

GO term	*p*-value	Number of proteins
*P. xylostella*		
Nucleic acid binding [GO:0003676]	5.78E-06	149
Oxidoreductase activity [GO:0016491]	1.64E-05	16
Oxidation-reduction process [GO:0055114]	0.0005938	38
Serine-type endopeptidase activity [GO:0004252]	0.001488	54
Protein tyrosine phosphatase activity [GO:0004725]	0.003778	12
Protein dephosphorylation [GO:0006470]	0.005278	14
Structural constituent of cuticle [GO:0042302]	0.006409	3
Zinc ion binding [GO:0008270]	0.008232	151
*M. sexta*		
Prothoracicotrophic hormone activity [GO:0018445]	2.99E-06	10
Growth factor activity [GO:0008083]	0.0026	5
Phosphorylase kinase complex [GO:0005964]	0.003736	3
SWI/SNF complex [GO:0016514]	0.003736	3
Phosphorylase kinase activity [GO:0004689]	0.003736	3
Phosphoprotein phosphatase activity [GO:0004721]	0.00556	6
Neuropeptide signaling pathway [GO:0007218]	0.007097	13
Defense response [GO:0006952]	0.00955	3
*H. melpomene*		
ATP-dependent peptidase activity [GO:0004176]	0.002622	2
Misfolded or incompletely synthesized protein catabolic process [GO:0006515]	0.002622	2
DNA integration [GO:0015074]	0.008793	2
Inositol-1,4,5-trisphosphate 3-kinase activity [GO:0008440]	0.008793	2
*D. plexippus*		
Dephosphorylation [GO:0016311]	0.002541	12
RNA-directed DNA polymerase activity [GO:0003964]	0.002617	16
Glucuronosyltransferase activity [GO:0015020]	0.003223	10
Endonuclease activity [GO:0004519]	0.004409	14
Carbohydrate transport [GO:0008643]	0.005052	12
Pyrophosphatase activity [GO:0016462]	0.008074	4
Riboflavin metabolic process [GO:0006771]	0.009158	8
Rho guanyl-nucleotide exchange factor activity [GO:0005089]	0.00975	10
*B. mori*		
Heme binding [GO:0020037]	1.30E-06	13
Monooxygenase activity [GO:0004497]	2.09E-06	13
Hormone activity [GO:0005179]	0.0001028	6
Electron transport [GO:0006118]	0.0003995	18
Calcium ion binding [GO:0005509]	0.00205	10
Response to oxidative stress [GO:0006979]	0.003103	3
Odorant binding [GO:0005549]	0.003145	6
Oxidoreductase activity [GO:0016491]	0.00358	16

In *M. sexta*, we identified a GO enrichment of prothoracicotrophic hormone activity (GO: 0018445). The prothoracicotropic hormone (PTTH) is well studied in tobacco hornworm (Rountree and Bollenbacher 1986) and in *M. sexta*, it is related to molting and metamorphosis [39, 40]. In *D. plexippus*, we identified the GO enrichment of glucuronosyltransferase activity (GO: 0015020). In silkworm, UDP-glucuronosyltransferase (UGT) plays a role in detoxification processes, such as minimizing the harmful effects of ingested plant allelochemicals [41]. Also, we identified the enrichment of Rho guanyl-nucleotide exchange factor activity (GO: 0005089), which is a modulator in the signaling pathway of Ras/MAPK and Wnt. Previous studies have shown that this activity is associated with neuronal growth cone and planar cell polarity formation [42, 43]. In *B. mori*, consistent with [30], we identified the enrichment of monooxygenase activity (GO: 0004497), which might be associated with detoxification.

To further clarify the functions of SDs in each Lepidoptera species, we annotated the gene functions in the SDs regions using Pfam and although the GO enrichments differed among species, some of the gene families embedded in the SDs were the same for the five species (Additional file 5: Table S5). For example, genes in SDs can be classified into three categories: (1) detoxification, (2) immunity, and (3) environmental signal recognition, which are similar to other mammals and insects [30, 44]. These genes are very important in drug detoxification, defense, and receptor and signal reorganization. The cytochrome P450s (P450s), for example, are important proteins for insect growth and development and have been found to play various functions such as biosynthesis of hormones, and inactivation and metabolism of xenobiotic compounds such as pesticides [45–47]. In this study, P450s were identified in all five species (8, 13, 15, 12, 10 SDs regions in *P. xylostella*, *B. mori*, *M. sexta*, *D. plexippus* and *H. melpomene,* respectively). In *P. xylostella*, Yu et al. [48] report strong expression of 84 functional cytochrome P450 genes, many of them, especially CYP367s, contributing to detoxification or metabolic processing of environmental chemicals.

We also identified the trypsin in the five species (55, 7, 24, 19, 2 SDs regions in *P. xylostella*, *B. mori*, *M. sexta*, *D. plexippus* and *H. melpomene,* respectively), which may be involved in immunity [49]. The glucose-methanol-choline (GMC) oxidoreductases, shown to be involved in developmental and physiological processes, and immunity [50], were also identified in four of the five species (1, 4, 5, 4 SDs regions in *B. mori*, *M. sexta*, *D. plexippus* and *H. melpomene,* respectively).

Some species-specific genes in SDs regions were also identified including 13 Lepidopteran-specific Lipoprotein_11 in silkworm. Zhang et al. [51] have shown that this family is involved in various physiological processes such as energy storage, embryonic development and immunity. These SDs might have played a role in the silkworm-specific evolution. Some lineage-specific expansion genes were also embedded in the SDs regions. For example, we identified 167 zinc-finger proteins in the SD regions of *P. xylostella*, which was much more than in any other species (20, 73, 91 and 8 in *B. mori*, *M. sexta*, *D. plexippus* and *H. melpomene*). A recent study (data unpubl.) of transcription factors in diamondback moth indicates that zinc-finger proteins may be expanded, also suggesting their potential important functions in the DBM. The zinc-finger has been shown to function in a variety of biological processes, such as DNA-binding, RNA-binding, protein-protein interactions, developmental processes and differentiation [52]. Further studies on expression patterns showed that the expression of some zinc-fingers were significantly different between susceptible and resistant strains (data unpublished). However, more researches are needed to illustrate the functions of these zinc-fingers.

Zhao et al. [30] report in silkworm that SDs are characterized by enrichment of DNA transposons and LTR retrotransposons. These observed enrichments in the flanking regions of SDs in silkworm suggest a potential implication in the formation of repeats in SDs. In this study, the TEs composition was analyzed by comparing the sequences near the SDs regions and found that DNA transposons were enriched in SDs regions as well as flanking regions of most species except *H. melpomene* (Table 3). Like in silkworm, DNA transposons and LTR (long terminal repeat) retrotransposons were enriched in the region of SDs and flanking regions in *P. xylostella* (Table 3), suggesting similar potential roles in SD formation. In *M. sexta* and *D. plexippus*, only DNA transposons were found to be enriched (Table 3). In *H. melpomene,* all analyzed TEs, except DNA transposons, were enriched in the SDs and flanking regions (Table 3) with LINEs (long interspersed nuclear elements) being the most abundant. These results suggest that short interspersed nuclear elements (SINEs), LTR and LINEs may also be involved in the formation of SDs in the genome of *H. melpomene*.

**Table 3:** TEs properties of the Lepidoptera genomes, duplications and 2.5 Kb flanking regions

Repeat	Duplication	%	2.5 Kb FR	%	Genome	%	Enrichment in SDs	Enrichment in FR
*P. xylostella*								
**DNA**	**72,462**	**0.167**	**55,718**	**0.053**	**180,461**	**0.046**	**3.653**	**1.152**
SINE	3040	0.007	22,679	0.021	258,493	0.066	0.107	0.327
**LTR**	**18,614**	**0.043**	**10,333**	**0.010**	**23,611**	**0.006**	**7.173**	**1.632**
LINE	249,583	0.576	129,227	0.122	628,430	0.159	3.613	0.767
*M. sexta*								
**DNA**	**11,814**	**0.062**	**22,773**	**0.045**	**128,714**	**0.031**	**2.015**	**1.462**
SINE	361	0.002	2714	0.005	48,452	0.011	0.164	0.463
LTR	131	0.001	1537	0.003	12,658	0.003	0.227	1.004
LINE	18,312	0.096	19,895	0.039	156,948	0.037	2.561	1.048
*D. plexippus*								
**DNA**	**12,322**	**0.052**	**22,852**	**0.044**	**73,287**	**0.029**	**1.779**	**1.484**
SINE	2783	0.012	4447	0.009	21,783	0.009	1.352	0.972
LTR	555	0.002	1975	0.004	7333	0.003	0.800	1.282
LINE	6026	0.026	4729	0.009	57,808	0.023	1.103	0.389
*H. melpomene*								
DNA	483	0.001	20,348	0.017	51,129	0.019	0.064	0.938
**SINE**	**23,660**	**0.058**	**15,872**	**0.014**	**26,036**	**0.009**	**6.169**	**1.436**
**LTR**	**7735**	**0.019**	**14,801**	**0.013**	**12,676**	**0.004**	**4.143**	**2.751**
**LINE**	**171,528**	**0.423**	**129,144**	**0.111**	**243,145**	**0.088**	**4.789**	**1.251**

*DNA* DNA transposons, *SINE* short interspersed nuclear elements, *LTR* long terminal repeat, *LINE* long interspersed nuclear elements

The TEs contents of three regions of the genomes were compared: SDs regions; 2.5 Kb flanking regions (FR) of the SDs and the genome average. Enrichment was defined as the repeat content of duplicated sequences divided by the repeat content of unique sequences. The significance was performed by simulating the repeats in a random sample (*n* = 1,00) of DBM SDs (*P*-value < 0.05 were in bold)

### Effects of SDs on gene expression

**Fig. 4:**
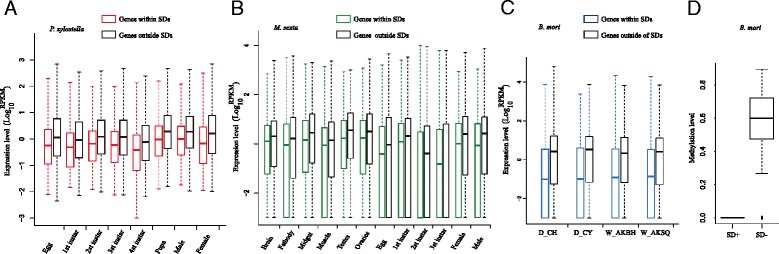
Expression levels are associated with segmental duplications in the 5 species. **a** Expression level analysis in *P. xylostella*. Different developmental stages (from egg to adult) are listed. The expression level for the genes located in SDs is marked as *red* while the expression level for the genes outside SDs is marked as *black*. **b** Expression level analysis in *M. sexta*. Different tissues and developmental stages used in the analysis are listed. The expression level for the genes located in SDs is marked as green while the expression level for the genes outside SDs is marked as *black*. **c** Expression level analysis in silk gland of *B. mori*. Different strains were analyzed including domesticated strain Chunhua (D_CH), domesticated strain Chunyu (D_CY), wild silkworm Ankang from Baihe county of Shanxi Province (W_AKBH) and wild silkworm Ankang from Shiquan county of Shanxi Province (W_AKSQ). **d** Methylation level comparison between genes within and without SDs regions based on silkworm data

An initial study of lymphoblastoid cell lines in human has shown that CNVs have some effects on gene expression [53]. For example, changes in the number of copies can explain almost 20% of the variation in gene expression [53]. This effect can be the results of gene dosage within SDs or SDs on neighboring genes [53–56]. To assess the effect of SDs on the transcriptomes, we explored the genome-wide expression of three of the Lepidoptera species, *P*. *xylostella*, *B. mori*, and *M. sexta*, at different developmental stages, different tissues and different strains using RNA-seq data (NCBI website). We found that the gene expression levels embedded within our SDs regions were significantly lower than that of other genes located elsewhere in the genome. This was true for all analyzed available developmental stages or tissues (T-test, *p* < 2.20E-16) (Fig. 4). For example, in *P*. *xylostella*, we analyzed the expression pattern of genes within and outside the SD regions in different developmental stages. The results showed that the expression values of genes within SDs were significantly lower than the genes outside the SDs regions (T-test, *p* < 0.01, Fig. 4a). We redid the same analysis on the silk gland from different strains of *B. mori* and different tissues of *M. sexta* and found similar expression patterns: genes located in SDs had lower expression values than the genes outside SDs (Fig. 4b and c).

A possible reason for this may be that some regulation mechanisms control the gene expression within SDs. Based on our analysis above, we found that some TEs were enriched in the SDs as well as the SDs’ flanking regions (Table 3). In silkworm, methylation levels in TEs regions are extremely low compared to the rest of the genome [57]. Epigenetic regulation in insects can have various effects on biological processes. In silkworm, CG methylation is enriched in gene bodies and is positively correlated with gene expression level, indicating its positive roles in gene transcription [57]. We therefore analyzed the CG methylation level of the genes embedded in the SD regions as well as the genes outside the SDs regions for the five species and did not find any CG methylation in gene bodies of SDs (Fig. 4c). This may explain the low gene expression levels in SDs regions. However, more CG methylation information from other Lepidoptera species may be needed to further validate this conclusion.

## Conclusion

Structural variation between genomes is important in phenotype differentiation and genome evolution. Here, we performed a comparative analysis of segmental duplications (SDs) among five lepidopteran reference genomes (*P. xylostella*, *D. plexippus*, *B. mori*, *M. sexta* and *H. melpomene*). We found that the SDs contents greatly varied among the five species. Comparative analyses of SDs showed that most of them arose after the divergence of each lineage. The most closely related species based on the phylogenetic tree also shared more common SDs. Conserved ancestral SDs and species specific SD events were assessed, revealing multiple examples of gain, loss or maintenance of SDs over time. The results indicated that SDs might have undergone loss or gain during the evolution of the genome. We further analyzed the genes embedded in SDs regions and the result showed that most of the genes were located in the species-specific SDs (“Unique” SDs). Functional analysis of these genes suggested their potential roles in the lineage-specific evolution. Comparison of gene expression between SDs and non-SDs showed that the expression levels of genes embedded in SDs were significantly lower, suggesting that structural changes in the genomes were involved in gene expression differences within each species. Our results suggested that SDs might have been involved in the species-specific evolution. They thus provide a valuable resource beyond the genetic mutation to explore the genome structure for future Lepidoptera research.

## Additional files

Additional file 1: Table S1. SDs in the five studied genomes. (XLSX 1727 kb)

Additional file 2: Table S2. Genes embedded in the SDs regions. (XLSX 81 kb)

Additional file 3: Table S3. Gene duplicates located in the SD intervals. (XLSX 68 kb)

Additional file 4: Table S4. GO enrichment for proteins within the SDs regions among the five studied Lepidoptera species. Only *p* < 0.01 were shown. (DOCX 20 kb)

Additional file 5: Table S5. Gene families that were found to be different among the SDs regions from the five studied genomes. (XLSX 10 kb)

## Abbreviations

CNV: Copy number variations;
GO: Gene Ontology
SD: Segmental duplications
SSR: Simple sequence repeats
TE: Transposable elements
WGAC: Whole-Genome Assembly Comparison

## References

[1] Marques-Bonet T, Girirajan S, Eichler EE (2009). The origins and impact of primate segmental duplications. Trends Genet.

[2] Duda TF, Palumbi SR (1999). Molecular genetics of ecological diversification: duplication and rapid evolution of toxin genes of the venomous gastropod Conus. Proc Natl Acad Sci U S A.

[3] Lynch M, Conery JS (2000). The evolutionary fate and consequences of duplicate genes. Science.

[4] Conant GC, Wolfe KH (2008). Turning a hobby into a job: how duplicated genes find new functions. Nat Rev Genet.

[5] Albano F, Anelli L, Zagaria A, Coccaro N, D'Addabbo P, Liso V, Rocchi M, Specchia G (2010). Genomic segmental duplications on the basis of the rearrangement in chronic myeloid leukemia. Oncogene.

[6] Marques-Bonet T, Kidd JM, Ventura M, Graves TA, Cheng Z, Hillier LW, Jiang Z, Baker C, Malfavon-Borja R, Fulton LA (2009). A burst of segmental duplications in the genome of the African great ape ancestor. Nature.

[7] Goidts V, Cooper DN, Armengol L, Schempp W, Conroy J, Estivill X, Nowak N, Hameister H, Kehrer-Sawatzki H (2006). Complex patterns of copy number variation at sites of segmental duplications: an important category of structural variation in the human genome. Hum Genet.

[8] Sharp AJ, Locke DP, McGrath SD, Cheng Z, Bailey JA, Vallente RU, Pertz LM, Clark RA, Schwartz S, Segraves R (2005). Segmental duplications and copy-number variation in the human genome. Am J Hum Genet.

[9] Bailey JA, Gu Z, Clark RA, Reinert K, Samonte RV, Schwartz S, Adams MD, Myers EW, Li PW, Eichler EE (2002). Recent segmental duplications in the human genome. Science.

[10] Cheng Z, Ventura M, She X, Khaitovich P, Graves T, Osoegawa K, Church D, DeJong P, Wilson RK, Paabo S (2005). A genome-wide comparison of recent chimpanzee and human segmental duplications. Nature.

[11] Kim PM, Lam HY, Urban AE, Korbel JO, Affourtit J, Grubert F, Chen X, Weissman S, Snyder M, Gerstein MB (2008). Analysis of copy number variants and segmental duplications in the human genome: Evidence for a change in the process of formation in recent evolutionary history. Genome Res.

[12] She X, Jiang Z, Clark RA, Liu G, Cheng Z, Tuzun E, Church DM, Sutton G, Halpern AL, Eichler EE (2004). Shotgun sequence assembly and recent segmental duplications within the human genome. Nature.

[13] She X, Cheng Z, Zollner S, Church DM, Eichler EE (2008). Mouse segmental duplication and copy number variation. Nat Genet.

[14] Umemori J, Mori A, Ichiyanagi K, Uno T, Koide T (2013). Identification of both copy number variation-type and constant-type core elements in a large segmental duplication region of the mouse genome. BMC Genomics.

[15] Nicholas TJ, Cheng Z, Ventura M, Mealey K, Eichler EE, Akey JM (2009). The genomic architecture of segmental duplications and associated copy number variants in dogs. Genome Res.

[16] You M, Yue Z, He W, Yang X, Yang G, Xie M, Zhan D, Baxter SW, Vasseur L, Gurr GM (2013). A heterozygous moth genome provides insights into herbivory and detoxification. Nat Genet.

[17] Duan J, Li R, Cheng D, Fan W, Zha X, Cheng T, Wu Y, Wang J, Mita K, Xiang Z (2009). SilkDB v2.0: a platform for silkworm (*Bombyx mori*) genome biology. Nucleic Acids Res.

[18] Consortium THG (2012). Butterfly genome reveals promiscuous exchange of mimicry adaptations among species. Nature.

[19] Zhan S, Reppert SM (2012). MonarchBase: the monarch butterfly genome database. Nucleic Acids Res.

[20] Xu HE, Zhang HH, Xia T, Han MJ, Shen YH, Zhang Z (2013). BmTEdb: a collective database of transposable elements in the silkworm genome. Database.

[21] Zhan S, Merlin C, Boore JL, Reppert SM (2011). The monarch butterfly genome yields insights into long-distance migration. Cell.

[22] Gotz S, Garcia-Gomez JM, Terol J, Williams TD, Nagaraj SH, Nueda MJ, Robles M, Talon M, Dopazo J, Conesa A (2008). High-throughput functional annotation and data mining with the Blast2GO suite. Nucleic Acids Res.

[23] Finn RD, Mistry J, Tate J, Coggill P, Heger A, Pollington JE, Gavin OL, Gunasekaran P, Ceric G, Forslund K (2010). The Pfam protein families database. Nucleic Acids Res.

[24] Fang SM, Hu BL, Zhou QZ, Yu QY, Zhang Z (2015). Comparative analysis of the silk gland transcriptomes between the domestic and wild silkworms. BMC Genomics.

[25] Whittington E, Zhao Q, Borziak K, Walters JR, Dorus S (2015). Characterisation of the *Manduca sexta* sperm proteome: Genetic novelty underlying sperm composition in Lepidoptera. Insect Biochem Mol Biol.

[26] Trapnell C, Pachter L, Salzberg SL (2009). TopHat: discovering splice junctions with RNA-Seq. Bioinformatics.

[27] Trapnell C, Roberts A, Goff L, Pertea G, Kim D, Kelley DR, Pimentel H, Salzberg SL, Rinn JL, Pachter L (2012). Differential gene and transcript expression analysis of RNA-seq experiments with TopHat and Cufflinks. Nat Protoc.

[28] Emanuel BS, Shaikh TH (2001). Segmental duplications: an ‘expanding’ role in genomic instability and disease. Nat Rev Gene.

[29] Fiston-Lavier AS, Anxolabehere D, Quesneville H (2007). A model of segmental duplication formation in *Drosophila melanogaster*. Genome Res.

[30] Zhao Q, Zhu Z, Kasahara M, Morishita S, Zhang Z (2013). Segmental duplications in the silkworm genome. BMC Genomics.

[31] Eichler EE (2001). Segmental duplications: what's missing, misassigned, and misassembled--and should we care?. Genome Res.

[32] Bailey JA, Eichler EE (2006). Primate segmental duplications: crucibles of evolution, diversity and disease. Nat Rev Gene.

[33] Osanai-Futahashi M, Suetsugu Y, Mita K, Fujiwara H (2008). Genome-wide screening and characterization of transposable elements and their distribution analysis in the silkworm, *Bombyx mori*. Insect Biochem Mol Biol.

[34] Sun W, Shen YH, Han MJ, Cao YF, Zhang Z (2014). An adaptive transposable element insertion in the regulatory region of the EO gene in the domesticated silkworm, *Bombyx mori*. Mol Biol Evol.

[35] Regier JC, Zwick A, Cummings MP, Kawahara AY, Cho S, Weller S, Roe A, Baixeras J, Brown JW, Parr C (2009). Toward reconstructing the evolution of advanced moths and butterflies (Lepidoptera: Ditrysia): an initial molecular study. BMC Evol Biol.

[36] Xia Q, Guo Y, Zhang Z, Li D, Xuan Z, Li Z, Dai F, Li Y, Cheng D, Li R (2009). Complete resequencing of 40 genomes reveals domestication events and genes in silkworm (Bombyx). Science.

[37] de la Paz C-MM, Wheat CW, Vogel H, Soderlind L, Janz N, Nylin S (2013). Mechanisms of macroevolution: polyphagous plasticity in butterfly larvae revealed by RNA-Seq. Mol Ecol.

[38] Hughes J, Vogler AP (2006). Gene expression in the gut of keratin-feeding clothes moths (Tineola) and keratin beetles (Trox) revealed by subtracted cDNA libraries. Insect Biochem Mol Biol.

[39] Rountree DB, Bollenbacher WE (1986). The release of the prothoracicotropic hormone in the tobacco hornworm, *Manduca sexta*, is controlled intrinsically by juvenile hormone. J Exp Biol.

[40] Riddiford L, Hiruma K, Zhou X, Nelson C (2003). Insights into the molecular basis of the hormonal control of molting and metamorphosis from *Manduca sexta* and *Drosophila melanogaster*. Insect Biochem Mol Biol.

[41] Luque T, Okano K, O'Reilly DR (2002). Characterization of a novel silkworm (*Bombyx mori*) phenol UDP-glucosyltransferase. Eur J Biochem.

[42] Morrison DK (2001). KSR: a MAPK scaffold of the Ras pathway?. J Cell Sci.

[43] Yin A, Pan L, Zhang X, Wang L, Yin Y, Jia S, Liu W, Xin C, Liu K, Yu X (2015). Transcriptomic study of the red palm weevil Rhynchophorus ferrugineus embryogenesis. Insect Sci.

[44] Liu GE, Ventura M, Cellamare A, Chen L, Cheng Z, Zhu B, Li C, Song J, Eichler EE (2009). Analysis of recent segmental duplications in the bovine genome. BMC Genomics.

[45] Scott JG (1999). Cytochromes P450 and insecticide resistance. Insect Biochem Mol Biol.

[46] Bernhardt R (2006). Cytochromes P450 as versatile biocatalysts. J Biotechnol.

[47] Iga M, Kataoka H (2012). Recent studies on insect hormone metabolic pathways mediated by cytochrome P450 enzymes. Biol Pharm Bull.

[48] Yu L, Tang W, He W, Ma X, Vasseur L, Baxter SW, Yang G, Huang S, Song F, You M (2015). Characterization and expression of the cytochrome P450 gene family in diamondback moth, *Plutella xylostella* (L.). Sci Rep.

[49] Kanost M, Gorman M. Phenoloxidases in insect immunity. In: Beckage N.E. (Ed.), Insect Immunol. Oxford: Academic Press; 2008.

[50] Sun W, Shen YH, Yang WJ, Cao YF, Xiang ZH, Zhang Z (2012). Expansion of the silkworm GMC oxidoreductase genes is associated with immunity. Insect Biochem Mol Biol.

[51] Zhang Y, Dong Z, Liu S, Yang Q, Zhao P, Xia Q (2012). Identification of novel members reveals the structural and functional divergence of lepidopteran-specific Lipoprotein_11 family. Funct Integr Genomic.

[52] Munoz-Descalzo S, Terol J, Paricio N (2005). Cabut, a C2H2 zinc finger transcription factor, is required during Drosophila dorsal closure downstream of JNK signaling. Dev Biol.

[53] Stranger BE, Forrest MS, Dunning M, Ingle CE, Beazley C, Thorne N, Redon R, Bird CP, de Grassi A, Lee C (2007). Relative impact of nucleotide and copy number variation on gene expression phenotypes. Science.

[54] Merla G, Howald C, Henrichsen CN, Lyle R, Wyss C, Zabot MT, Antonarakis SE, Reymond A (2006). Submicroscopic deletion in patients with Williams-Beuren syndrome influences expression levels of the nonhemizygous flanking genes. Am J Hum Genet.

[55] Henrichsen CN, Vinckenbosch N, Zollner S, Chaignat E, Pradervand S, Schutz F, Ruedi M, Kaessmann H, Reymond A (2009). Segmental copy number variation shapes tissue transcriptomes. Nat Genet.

[56] Blekhman R, Oshlack A, Gilad Y (2009). Segmental duplications contribute to gene expression differences between humans and chimpanzees. Genetics.

[57] Xiang H, Zhu J, Chen Q, Dai F, Li X, Li M, Zhang H, Zhang G, Li D, Dong Y (2010). Single base-resolution methylome of the silkworm reveals a sparse epigenomic map. Nat Biotechnol.

