# Supplementary material for: Segmental duplications: evolution and impact among the current Lepidoptera genomes
Source: BMC Evol Biol. 2017 Jul 6;17:161. doi: 10.1186/s12862-017-1007-y (PMC5499213; doi:10.1186/s12862-017-1007-y)
Supplement: Supplementary file 4 — GO enrichment for proteins within the SDs regions among the five studied Lepidoptera species. Only p < 0.01 were shown. (DOCX 20 kb) [file 12862_2017_1007_MOESM4_ESM.docx]

Supplemental Table 4. GO enrichment for proteins within the SDs regions among the five Lepidoptera species.

| GO term* | p-value | Number of proteins |
| --- | --- | --- |
| ***P. xylostella*** | | |
| DNA integration [GO:0015074] | 1.32E-15 | 50 |
| RNA binding [GO:0003723] | 2.39E-13 | 90 |
| Helicase activity [GO:0004386] | 4.42E-12 | 59 |
| Nucleic acid binding [GO:0003676] | 5.78E-06 | 149 |
| Endopeptidase [GO:0004190] | 1.35E-05 | 16 |
| Oxidoreductase activity [GO:0016491] | 1.64E-05 | 16 |
| DNA binding [GO:0003677] | 5.24E-05 | 117 |
| Proteolysis [GO:0006508] | 7.60E-05 | 103 |
| Regulation of transcription, DNA-templated [GO:0006355] | 0.0003882 | 22 |
| Oxidation-reduction process [GO:0055114] | 0.0005938 | 38 |
| Metabolic process [GO:0008152] | 0.000603 | 52 |
| Transport [GO:0006810] | 0.0006568 | 37 |
| Chromatin [GO:0000786] | 0.0008083 | 12 |
| Nucleosome assembly [GO:0006334] | 0.001239 | 12 |
| Serine-type endopeptidase activity [GO:0004252] | 0.001488 | 54 |
| Protein tyrosine phosphatase activity [GO:0004725] | 0.003778 | 12 |
| Carbohydrate metabolic process [GO:0005975] | 0.004171 | 11 |
| Protein dephosphorylation [GO:0006470] | 0.005278 | 14 |
| RNA-DNA hybrid ribonuclease activity [GO:0004523] | 0.005709 | 6 |
| Transporter activity [GO:0005215] | 0.005726 | 23 |
| Structural constituent of cuticle [GO:0042302] | 0.006409 | 3 |
| Zinc ion binding [GO:0008270] | 0.008232 | 151 |
| Fatty-acyl-CoA binding [GO:0000062] | 0.008344 | 5 |
| Dephosphorylation [GO:0016311] | 0.009491 | 12 |
| ***M. sexta*** | | |
| Prothoracicotrophic hormone activity [GO:0018445] | 2.99E-06 | 10 |
| Extracellular region [GO:0005576] | 0.0008552 | 25 |
| DNA binding [GO:0003677] | 0.001266 | 35 |
| Extracellular space [GO:0005615] | 0.001954 | 10 |
| Binding [GO:0005488] | 0.00231 | 20 |
| Growth factor activity [GO:0008083] | 0.0026 | 5 |
| Growth [GO:0040007] | 0.0026 | 5 |
| cell proliferation [GO:0008283] | 0.0026 | 5 |
| Structural constituent of cuticle [GO:0042302] | 0.003576 | 19 |
| Phosphorylase kinase complex [GO:0005964] | 0.003736 | 3 |
| SWI/SNF complex [GO:0016514] | 0.003736 | 3 |
| Phosphorylase kinase activity [GO:0004689] | 0.003736 | 3 |
| ATP-dependent chromatin remodeling [GO:0043044] | 0.00431 | 4 |
| Phosphoprotein phosphatase activity [GO:0004721] | 0.00556 | 6 |
| Neuropeptide signaling pathway [GO:0007218] | 0.007097 | 13 |
| Glycogen biosynthetic process [GO:0005978] | 0.00955 | 3 |
| Defense response [GO:0006952] | 0.00955 | 3 |
| ***H. melpomene*** | | |
| ATP-dependent peptidase activity [GO:0004176] | 0.002622 | 2 |
| Misfolded or incompletely synthesized protein catabolic process [GO:0006515] | 0.002622 | 2 |
| DNA integration [GO:0015074] | 0.008793 | 2 |
| Inositol-1,4,5-trisphosphate 3-kinase activity [GO:0008440] | 0.008793 | 2 |
| ***D. plexippus*** | | |
| Ribosome [GO:0005840] | 0.002027 | 8 |
| Dephosphorylation [GO:0016311] | 0.002541 | 12 |
| RNA-dependent DNA replication [GO:0006278] | 0.002617 | 16 |
| RNA-directed DNA polymerase activity [GO:0003964] | 0.002617 | 16 |
| Glucuronosyltransferase activity [GO:0015020] | 0.003223 | 10 |
| Nuclear pore [GO:0005643] | 0.003692 | 8 |
| Endonuclease activity [GO:0004519] | 0.004409 | 14 |
| Membrane [GO:0016020] | 0.004707 | 34 |
| Carbohydrate transport [GO:0008643] | 0.005052 | 12 |
| Acid phosphatase activity [GO:0003993] | 0.005962 | 8 |
| Hexachlorocyclohexane metabolic process [GO:0019497] | 0.006001 | 9 |
| Lipid metabolic process [GO:0006629] | 0.007432 | 8 |
| Nucleoside transmembrane transporter activity [GO:0005337] | 0.008074 | 4 |
| Spectrin [GO:0008091] | 0.008074 | 4 |
| Pyrophosphatase activity [GO:0016462] | 0.008074 | 4 |
| Riboflavin metabolic process [GO:0006771] | 0.009158 | 8 |
| Regulation of Rho protein signal transduction [GO:0035023] | 0.00975 | 10 |
| Rho guanyl-nucleotide exchange factor activity [GO:0005089] | 0.00975 | 10 |
| ***B. mori*** | | |
| Extracellular region [GO:0005576] | 5.58E-12 | 27 |
| Heme binding [GO:0020037] | 1.30E-06 | 13 |
| Monooxygenase activity [GO:0004497] | 2.09E-06 | 13 |
| Phospholipid metabolic process [GO:0006644] | 2.88E-06 | 6 |
| Iron ion binding [GO:0005506] | 4.86E-06 | 14 |
| Phospholipase A2 activity [GO:0004623] | 6.03E-06 | 6 |
| Hormone activity [GO:0005179] | 0.0001028 | 6 |
| Electron transport [GO:0006118] | 0.0003995 | 18 |
| Acid phosphatase activity [GO:0003993] | 0.001512 | 4 |
| Catalase activity [GO:0004096] | 0.00176 | 3 |
| Calcium ion binding [GO:0005509] | 0.00205 | 10 |
| Response to oxidative stress [GO:0006979] | 0.003103 | 3 |
| Odorant binding [GO:0005549] | 0.003145 | 6 |
| Oxidoreductase activity [GO:0016491] | 0.00358 | 16 |
| metallopeptidase activity [GO:0008237] | 0.006056 | 3 |
| Metabolic process [GO:0008152] | 0.007134 | 17 |
| ATP BINDING [GO:0005524] | 0.009557 | 5 |

*Only *p* < 0.01 were listed in this table.
